# Enzymatic activities of proteins encoded by PmurB, PmurC, and PmurE involved in methanogen pseudomurein biosynthesis

**DOI:** 10.3389/fmicb.2026.1766147

**Published:** 2026-03-04

**Authors:** Guihong Cha, Zhenli Lai, Shuxin Wang, Qing Yang, Pengyan Zhao, Yi Chen, Wei Han, Liping Bai

**Affiliations:** 1 School of Food and Liquor Engineering (School of Wuliangye Baijiu), Sichuan University of Science and Engineering, Yibin, China; 2 Brewing Science and Technology Key Laboratory of Sichuan Province, Sichuan University of Science and Engineering, Yibin, Sichuan, China; 3 Key Laboratory of Southwest China Wildlife Resources Conservation (Ministry of Education), College of Life Science, China West Normal University, Nanchong, China; 4 Institute of Environmental Health and Ecological Security, School of Emergency Management, School of the Environment and Safety, Jiangsu University, Zhejiang, Jiangsu, China; 5 Key Laboratory of Development and Application of Rural Renewable Energy, Biogas Institute of the Ministry of Agriculture and Rural Affairs, Chengdu, Sichuan, China

**Keywords:** enzymatic activities, methanogens, PMur ligase, pseudomurein biosynthesis, structural

## Abstract

Pseudomurein, a glycan polymer present in the cell wall, is found in the orders Methanobacteriales and Methanopyrales. It possesses glycan units and peptide chains similar to those of bacterial peptidoglycan (murein). However, the biosynthesis of pseudomurein remains unknown. In this study, we identified and characterized several key enzymes, such as PMurBCE ligases, that are proposed to catalyse peptide chain biosynthesis. Specifically, PMurB catalyses the conjugation of UDP-Glu and Pi, forming UDP. PMurC then transfers alanine or threonine to UDP-Glu, and PMurE adds lysine to the PMurC product. These reactions represent the enzymatic activities of PMurBCE ligases in methanogens. Structural model analyses indicated that archaeal PMur enzymes share an overall structural arrangement similar to that of bacterial Mur ligases but exhibit specificity for different substrates. The structural model data provide insights into pseudomurein synthesis in methanogens and create promising avenues for biotechnological applications in methanogens, such as gene editing and the development of novel antibacterial agents targeting methanogenic archaea.

## 1 Introduction

Methanogens have received increased research interest because of their key role in climate change via methane emissions, with methane being a greenhouse gas (Reeburgh, 2007; Kirschke et al., 2013; Peng, 2023). Most atmospheric methane is produced by methanogenic archaea, which reduce simple substrates such as carbon dioxide, acetate, methylamine, and methanol to methane (Liu and Whitman, 2008; Lyu et al., 2018). Methanogens are widely distributed across diverse environments, including hot springs, seafloors, wetlands, and even animal digestive systems (Thauer et al., 2008). However, methanogens remain poorly understood due to challenges in genetic manipulation arising from their extreme growth conditions and unique cell wall composition. Common antibiotics and lysozymes are ineffective against methanogens, resulting in the absence of established gene-editing techniques and associated biotechnological applications.

**Figure 1:**
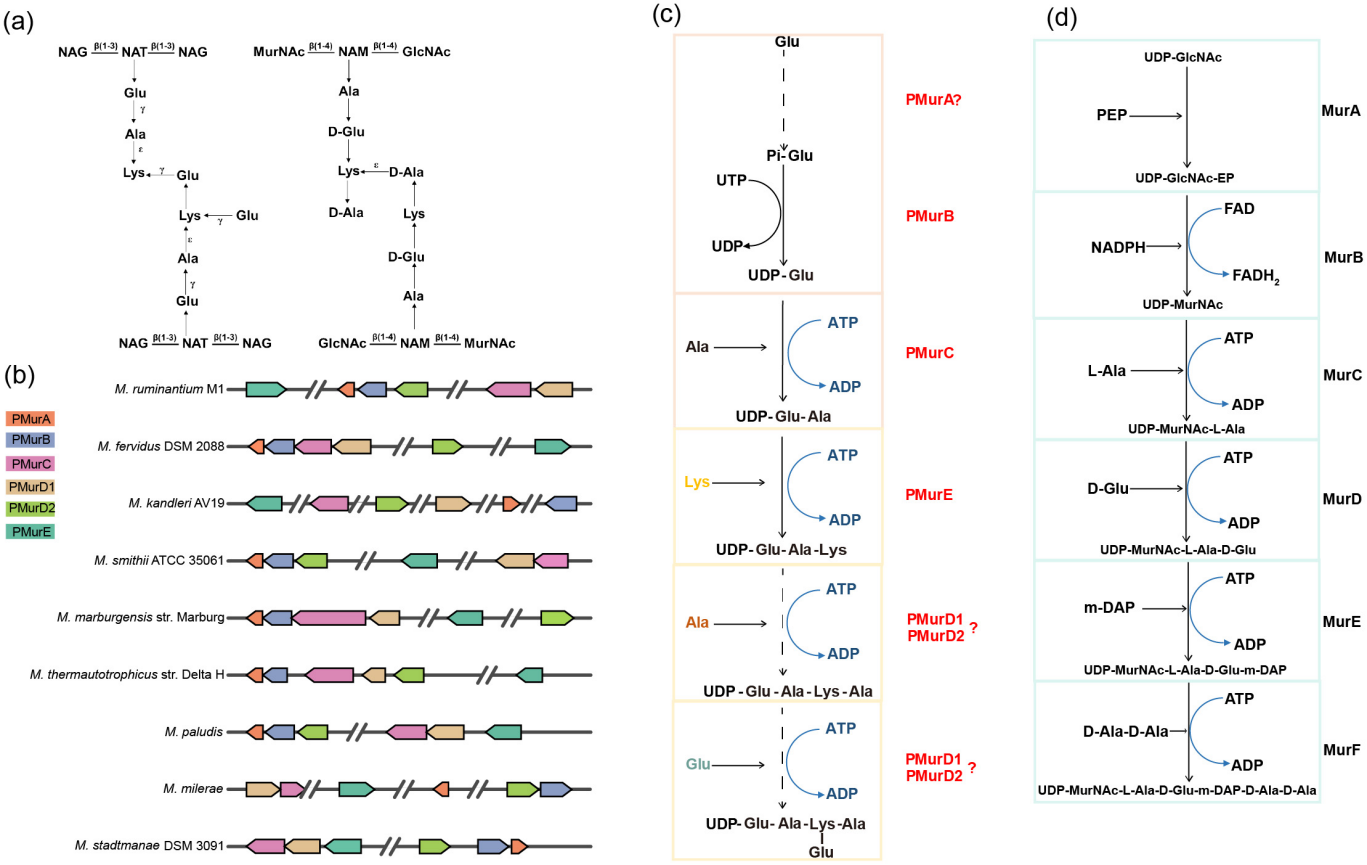
Pseudomurein biosynthesis gene clusters and predicted pathway: **(a)** Composition of pseudomurein structure and murein structure (Subedi et al., 2021); NAG, N-acetylglucosamine; NAT, N-acetyltalosaminuronic acid; MurNAc, N-acetylmuramic acid; GlcNAc, N-acetylglucosamine; **(b)** Gene clusters for pseudomurein biosynthesis in methanogens (Supplementary Table S2); **(c)** Predicted pseudomurein biosynthesis pathway; **(d)** Predicted murein biosynthesis pathway; PEP, phosphoenolpyruvate; UDP-GlcNAc, UDP-n-acetylglucosamine; UDP-MurNAc, UDP-n-acetylmuramic acid; m-DAP, *meso*-Diaminopimelic acid.

Methanogens possess diverse cell wall components, including S-layers, methanochondroitin, heteropolysaccharides, proteinaceous sheaths, and pseudomurein (Klingl et al., 2019). Pseudomurein is structurally similar to bacterial murein and is only found in Methanobacteriales and Methanopyrales (Subedi et al., 2021). In comparison to murein, research on pseudomurein biosynthesis in methanogens has been limited. Pseudomurein of methanogens is a structural analog of murein, comprising a peptide cross-linked glycan backbone, but its chemistry shows major differences (Figure 1a). These differences include the presence of a methanogen-specific aminosugar N-acetyl-L-talosaminuronic acid (NAT), β(1–3) bonds in the glycan back-bone and isopeptide bonds (ε and γ), and the presence of only L-amino acids in the peptide stem (KöNig et al., 1994; Hartmann and König, 1990; Leps et al., 1984). Pseudomurein peptide biosynthesis has been proposed to proceed through the biosynthesis of an unusual UDP-Nα-glutamyl-γ-phosphate precursor that is formed in three steps, not through UDP-N-acetylmuramic acid as observed in bacteria (Subedi et al., 2021; KöNig et al., 1994). During the peptide chain biosynthesis, amino acid residues are sequentially added into the UDP-glutamate intermediate following a similar model to murein. Most of the homologous proteins of murein peptide ligase MurA-F have been identified in methanogens (Basavannacharya et al., 2010; Leahy et al., 2010). For example, the homologous proteins of peptide ligases MurC, MurD, and MurE in bacteria are identified in pseudomurein containing methanogens, namely, PMurC, PMurD, and PMurE (Subedi et al., 2021, 2022). However, MurA and MurB, which catalyse the first two steps of murein biosynthesis, are absent in methanogens because the latter utilizes glutamate as the initial substrate rather than UDP-N-acetylglucosamine. Subedi et al. (2021, 2022) determined the crystal structures of the pseudomurein peptide ligases pMurC and pMurE, demonstrating their structural similarity to bacterial Mur proteins. However, the enzymatic function of these methanogen proteins and the reactions of pseudomurein peptide linkage have not yet been reported.

In this study, we elucidated the function of three key enzymes involved in pseudomurein pentapeptide biosynthesis through enzymatic and structural assays. Interestingly, an NTP transferase gene adjacent to PMurC, unique to methanogens, encodes PMurB, which catalyses the conjugation of UDP-Glu and Pi. Following Pi-Glu activation, PMurC and PMurE add alanine/threonine and lysine, respectively, to UDP-Glu, thereby extending the pentapeptide. Although sequence and structural analyses indicate that PMur enzymes are evolutionarily closely related to bacterial Mur ligases, they do not share the same functions as their bacterial counterparts. The elucidation of biological functions for Pmur ligases and peptide chain biosynthesis impacts biotechnological and agricultural applications involving biological methane production.

## 2 Materials and methods

### 2.1 Homologous protein search and cluster analysis

The sequences of bacterial MurC-E were used to search for homologous proteins sequences in *M. ruminantium* M1 and *M. fervidus* using TBtools with default settings (Chen et al., 2023). The sequences of PMur from *M. ruminantium* M1 were selected as query sequences for a BLASTP search (BLAST+2.15.0, e^−10^) in other pseudomurein-containing methanogens. Candidates were screened for domain organization by searching the SMART database (Letunic et al., 2020), and the picture of the gene cluster was visualized using ChiPlot (https://www.chiplot.online/). All pMurA-E homologous proteins are listed in Supplementary Tables S1–S3, S5. The multiple sequences were aligned with GENEDOC (Nicholas and Nicholas, 1997).

### 2.2 Chemical synthesis of UMP-Glu-Ala, UMP-Glu-Thr, and UMP-Glu-Ala-Lys

In a three-neck round-bottom flask, uridine5′-monophosphate (0.26 g, 0.774 mmol), L-Glu-Ala (1.34 g, 5.4 mmol), or L-Glu-Thr (1.42 g, 5.4 mmol) and Dicyclohexylcarbodiimide (1.6 g, 7.74 mmol) were dissolved in a mixture of t-BuOH and water (5:1, 40 ml). 1-Hydroxybenzotriazole (0.104 g, 0.774 mmol) was added to this mixture. The reaction mixture was heated at 85 °C for 4 h with stirring under argon. Upon completion, the reaction mixture was cooled to room temperature, and the solvent was removed with rotary evaporation while the water bath temperature was maintained below 30 °C. The product was isolated using TLC (i-PrOH/H_2_O/NH_3_, 20:1:0.5) and identified as the spot at the origin. This product was dissolved in 0.4 M NaOH in a MeOH/H_2_O mixture (4:1, 10 ml) and stirred at room temperature under argon for 8 h. The reaction progress was monitored using TLC (i-PrOH/H_2_O/NH_3_, 6:2:2) until the disappearance of the starting material. The reaction mixture was neutralized by adding triethylammonium acetate (2 M, pH 7.5). The product was isolated using TLC (i-PrOH/H_2_O/NH_3_, 6:2:2) and identified as the spot at the origin. Next, after subjecting the solution to magnetic stirring (~500 rpm) for 14 h at 55 °C, it was bubbled with nitrogen gas (99.999%) for 30 min at room temperature. To obtain pure UMP-linked peptide products, the mixture was passed through an HC-C18 SPE Cartridge (100 mg, 1 ml, ANPEL Lab Technologies) with 0.8 ml 80% acetonitrile–water solution as an eluent. All final UMP-linked peptide products were analyzed, and their structures were confirmed using 1H NMR and HRMS (Supplementary Table S4). The UMP-linked peptide products were kept at −20 °C until analysis.

### 2.3 Strains and cultivation

*Escherichia coli* DH5α and BL21(DE3) were grown in LB agar plates or LB broth at 37 °C and 180 rpm; BL21 Star (DE3) was grown in 2 × YT medium at 37 °C and 200 rpm; the final concentration of ampicillin or kanamycin used for selecting recombinant plasmids was 50 μg/ml.

### 2.4 Expression and purification of PMurA-E

Recombinant plasmids of PMurA-E were cloned into the *Xho*I and *Nco*I sites in the vector pET-28a (Novagen, USA) or pGEX-6p (Novagen, USA) and contained an N-terminal tag of 6 × His to form recombinant expression plasmids, and these vectors were synthesized by AZENT (Suzhou, China). More detailed information on expression strains, plasmids, and antibiotics is provided in Supplementary Table S2. The recombinant plasmids of PMurA-E were transformed into *E. coli* BL21 (DE3) or BL21 Star (DE3) to form a recombinant expression strain (Supplementary Table S2). The expression strains were cultured to OD_600_ = 0.6, then the final concentration of 0.1 mM IPTG was added to induce protein expression for 15 h with shaking at 16 °C and 120 rpm.

Cells were harvested (8,000 rpm, 15 min), resuspended with buffer A (300 mM NaCl, 10 mM imidazole, 50 mM Tris, pH 7.5), and sonicated on ice for 20 min (3/9 s ON/OFF) on an ice–water mixture. Subsequently, the mixture was centrifuged at 18,000 rpm for 30 min at 4 °C. The supernatant was filtered with a 0.22 μm filter and then loaded onto a His-Trap™ HP column, which was pre-equilibrated with five column volumes (CVs) of buffer A. After loading, the column was washed with 10 CVs of buffer A until no material appeared in the effluent. The target proteins were eluted with a gradient mixed with buffer B elution buffer (300 mM NaCl, 500 mM imidazole, 50 mM Tris, pH 7.5). The purified recombinant proteins were analyzed using Western blotting and SDS–PAGE (Supplementary Figures S2, S3), and the Bradford method was employed to calculate protein concentrations using bovine serum as a standard.

### 2.5 Enzymatic assays

The PMurA, PMurB, PMurC, PMurE, and PMurD1 activities for Glu, UMP-Glu, UMP-Glu-Ala, and UMP-Glu-Ala-Lys (like), respectively, were characterized using high-performance liquid chromatography (HPLC) and liquid mass spectrometry (LC-MS) on the corresponding products. The standard assay was performed at 37 °C for 12 h. The reaction mixture (5 ml) consisted of a buffer containing 10 mM Na_2_HPO_4_ and 1.8 mM KH_2_PO_4_ (pH 7.4), 137 mM NaCl, 2.7 mM KCl, and 5 μM MgCl_2_, supplemented with 1 mM ATP, 1 mM MgCl_2_, 20 μM of the pseudomurein synthesis pathway key enzymes (PMurA, PMurB, PMurC, and PMurE), and 1 mM of the substrate (Glu, UMP-Glu, and UMP-Glu-Ala). The reaction was initiated by the addition of the enzymes to the reaction mixture. The enzyme reactions were terminated by enzyme removal using a 3 kDa ultrafiltration tube at 4 °C and 10,000 rpm for 30 min. The solid was isolated by the rotary evaporation of the enzyme reaction solution using a vacuum concentrator; the isolated solid was resuspended in 200 μl ddH_2_O and filtered through a 0.22 μm membrane. ATP consumption was monitored using HPLC and LC-MS after placing the obtained filtrate in a liquid-phase vial with an inserted inner tube.

#### 2.5.1 HPLC assay

In the synthesis of pseudomurein peptide unit precursors, enzymes consume ATP to complete their reaction, and ATP absorbs at 254 nm (Bartlett and Lewis, 1970); thus, an enzyme's activity can be initially determined by detecting the consumption of ATP via LC-MS (Supplementary Figure S12) using reversed-phase fully porous silica C18 (1.7 μm, 2.1 × 150 mm, 75 cm, Thermo Fisher Scientific, USA); mobile phase: 0.1 M NH_4_H_2_PO_4_ (pH = 6.0) in 1% methanol (v/v); flow rate: 0.8 ml/min; injection volume: 20 μl; detection wavelength: 254 nm; and column temperature: 30 °C.

#### 2.5.2 LC-MS assay

LC-MS was used to test whether the key enzyme for the synthesis of the pseudomurein peptide unit precursor could catalyse the corresponding substrate analogs to produce the desired product and thus verify its biological function (Supplementary Table S3 and Supplementary Figure S1). Mobile phases: 90% acetonitrile (5–8 min), 90%−10% acetonitrile (8–8.1 min), and 10% acetonitrile (8.1–10 min); flow rate: 0.2 ml/min; ionization mode: negative; MS1: DP: −70; EP: −10; MS2: DP: −70; EP: −10; CE- 35; and CXP: −7.

### 2.6 Structural analysis and molecular substrate docking

The structures of PMurC-E were modeled and predicted using AphaFold2 (Mirdita et al., 2022). Substrate binding analyses of PMurB, PMurC, PMurD, and PMurE were performed using homology modeling. The quality of AlphaFold2-predicted structures was assessed using pLDDT scores, which indicated overall high confidence (average score >90). Homologs were identified, substrates were appropriately positioned, and the docked ligands were energy-minimized using UCSF Chimera (Pettersen et al., 2004). Ligand structures were built and energy-minimized using ChemDraw3D. Ligand positions within the pMur enzymes were determined via structural alignment with homologous bacterial Mur ligases. Docking simulations were then conducted using AutoDock Vina (Trott and Olson, 2010; Eberhardt et al., 2021), and a restricted search box was used to optimize ligand orientations. All docking results were visually inspected against protein surfaces to confirm the absence of steric clashes. Structures were visualized and exported as images using PyMOL (https://pymol.org/).

## 3 Results and discussion

### 3.1 Predicted genes and pathways of pseudomurein biosynthesis

**Figure 2:**
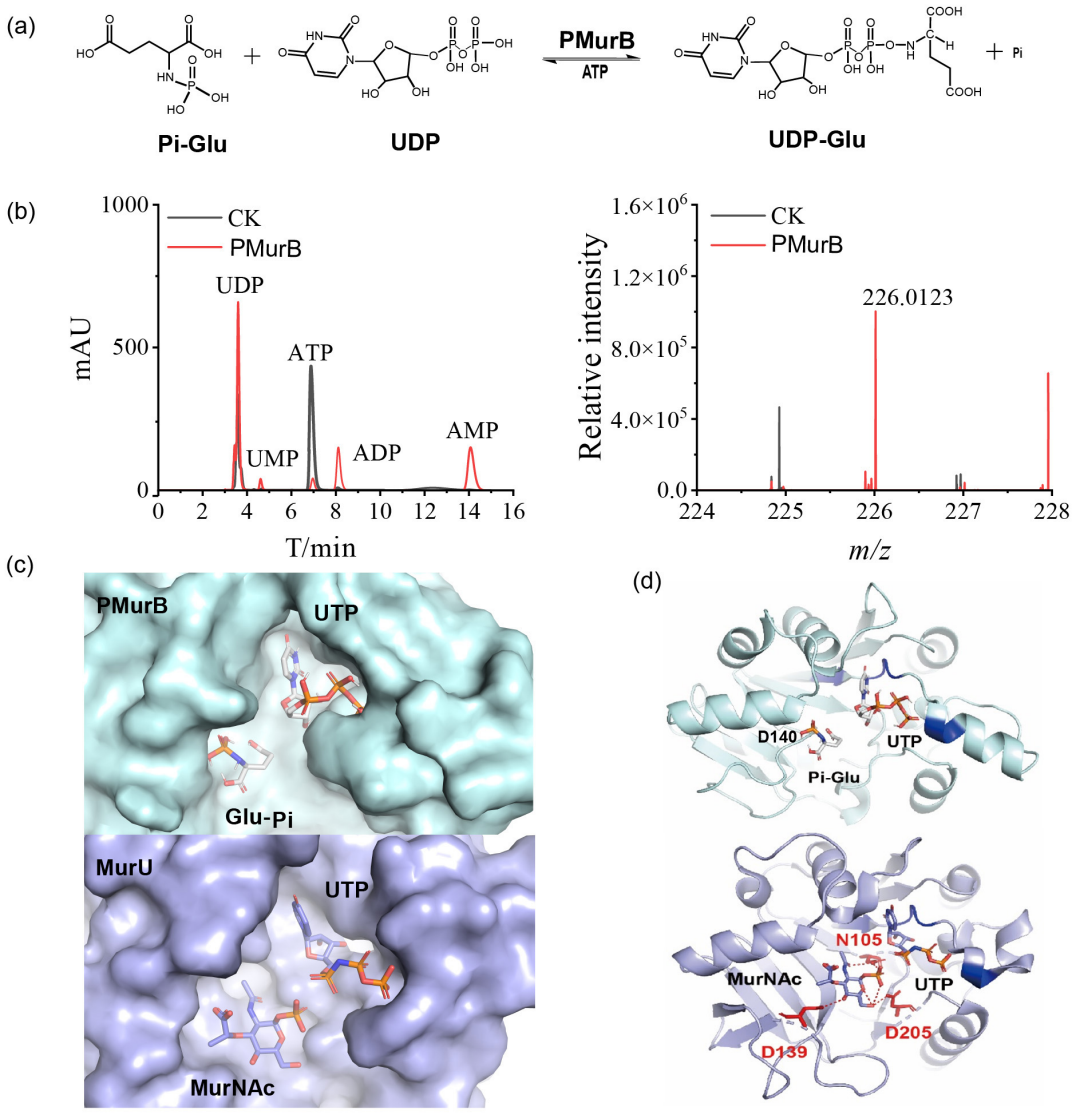
Activity and structural analysis of PMurB: **(a)** equation of reaction catalyzed by PMurB. **(b)** analysis of UTP synthesis capacity of PMurB with HPLC (left) and high-resolution detection of reverse reaction product N-P-Glu (right). **(c)** superposition alignment between PMurB and MurU (PDB 4Y7U, RMSD 1.863, conserved ATP-binding residues colored in blue), left; active chamber of PMurB (pale cyan) and MurU (light blue), right. **(d)** Structural analysis of MurU and PMurB: Down: MurU (PDB: 4Y7U) is shown in light blue, conserved NTP-binding domain is blue-colored, ligands are shown as sticks, and residues binding to MurNAc are marked and colored in red; Up: PMurB modeled using AlphaFold2 is shown in pale cyan, and highly conserved residues around UTP are blue-colored. HPLC analyses were performed in triplicate for each sample. CK: control check group (enzymatic reaction system proceeds without enzyme addition).

In bacteria, murein peptide chain biosynthesis is catalyzed by the conserved enzymes MurA-F (El Zoeiby et al., 2003). Homologs of MurA–F are present in methanogens, as determined by protein sequence alignments (Letunic et al., 2020). Bacteria synthesize UDP-N-acetylglucosamine from glucosamine-6-phosphate, which is derived from fructose-6-phosphate, a glycolytic intermediate. Two neighboring genes, PMurA and PMurB, co-cluster with the PMurC gene cluster in methanogens (Figure 1b). In detail, PMurB contained an NTP transferase domain and shared 24.49% identity with MurU, which generates UDP-MurNAc from UTP and MurMac-Pi in fosfomycin-resistant bacteria (Supplementary Table S1 and Supplementary Figure S5). Thus, it was hypothesized that PMurB catalyses the conjugation of UDP and Glu, forming UDP-Glu at the second step of pentapeptide biosynthesis, while PMurA catalyses glutamate phosphorylation since this protein contained a conserved NTPase domain (Supplementary Table S1). Specifically, PMurC, PMurD, and PMurE are found in all pseudomurein-producing methanogens and are clustered in the genome (Figure 1b). PMurC shared 20%−40% sequence identity with MurC, and its overall structure is highly similar to that of MurC, suggesting that PMurC catalyses a similar reaction to MurC (Figure 2c and Supplementary Figure S6). Therefore, PMurC was predicted to catalyse the ligation of alanine and UDP-Glu at the third step of pentapeptide biosynthesis (Figure 1c). Similarly, the structure of PMurE indicates that it performs the same function as MurE, i.e., adding lysine to the PMurC product UDP-Glu-Ala (Figures 1c, d). In murein biosynthesis, MurD adds glutamate to UDP-MurNAc-Ala. In pseudomurein-producing methanogens, two MurD-like proteins (PMurD1 and PMurD2) are present (Figure 1b). Thus, we propose that one of these PMurD homologs catalyses the addition of L-Ala and L-Glu residues in the final two steps of pentapeptide biosynthesis (Figure 1c).

### 3.2 pMurB is a UDP-transferase that generates UDP

According to the domain analysis, PMurB had an NTP transferase domain, and its overall structure, constructed by AlphaFold2, was similar to the bacterial MurU, which contributes to fosfomycin resistance in special Gram-negative bacteria like Pseudomonas (Figure 2c, Supplementary Table S1). Therefore, it was predicted that this protein primarily catalyses the formation of UDP from Pi and UDP-Glu (Figure 2a). To verify this hypothesis, PMurB from *M. ruminantium* was expressed and purified (Supplementary Figure S3a). The function of UDP synthesis was tested, and the HPLC results showed that UDP was generated (Figure 2b, left), demonstrating that PMurB catalyses the conjugation of UDP-Glu and ATP, forming UDP.

Due to the challenges in synthesizing the intermediate UDP-Glu, the structural analog UMP-Glu was employed to assess PMurB activity, as the diphosphate moiety of UDP cannot be reliably preserved during chemical synthesis (Supplementary Figure S1a, Supplementary Table S4). PMurB hydrolysed both UTP and ATP, producing Pi-Glu (m/z: 227.0194 Da), as confirmed with mass spectrometry (Figure 2b, right; Supplementary Figure S4a). Moreover, secondary fragments (C_5_H_8_O_4_)^−^ (*m/z*: 131.04 Da) and (H_4_NO_3_P)^−^ (*m/z*: 96.99 Da) were clearly detected, verifying that pMurB catalyses Glu-Pi formation and serves as a key enzyme in the pentapeptide biosynthesis pathway (Supplementary Figure S4b).

To elucidate the catalytic mechanism of PMurB, its structure was predicted using AlphaFold. The overall structure features a conserved N-terminal catalytic domain and a Rossmann fold containing a dinucleotide-binding region (Figure 2c). Dali server analysis indicated high structural similarity between pMurB and MurU (PDB: 4Y7U), as well as other nucleotidyltransferases (Subedi, 2018). Despite low sequence identity, these enzymes share similar catalytic domain organization and common structural features (Supplementary Figure S5 and Figure 2d).

In MurU, the substrate UTP and MurNAc were coordinated at distinct sites within the active site, a solvent-exposed cleft in the N-terminal core domain, where the sugar ring of MurNAc forms hydrogen bonds with various residues (Figure 4c, down). Molecular docking simulations revealed that, in PMurB, UTP occupies an active site pocket within a deep cleft formed by the central β-sheet and flexible loops, analogous to its positioning in MurU (Figure 4c, up). However, the glutamate-binding chamber in PMurB is substantially smaller than the sugar-binding chamber in MurU, rendering MurNAc unable to bind to PMurB. This structural difference may explain why PMurB and MurU cannot catalyse each other's reactions despite their structural similarities and why PMurB is exclusive to methanogens (Figure 2c).

### 3.3 PMurC catalyses the ligation of alanine or threonine to UDP-Glu

**Figure 3:**
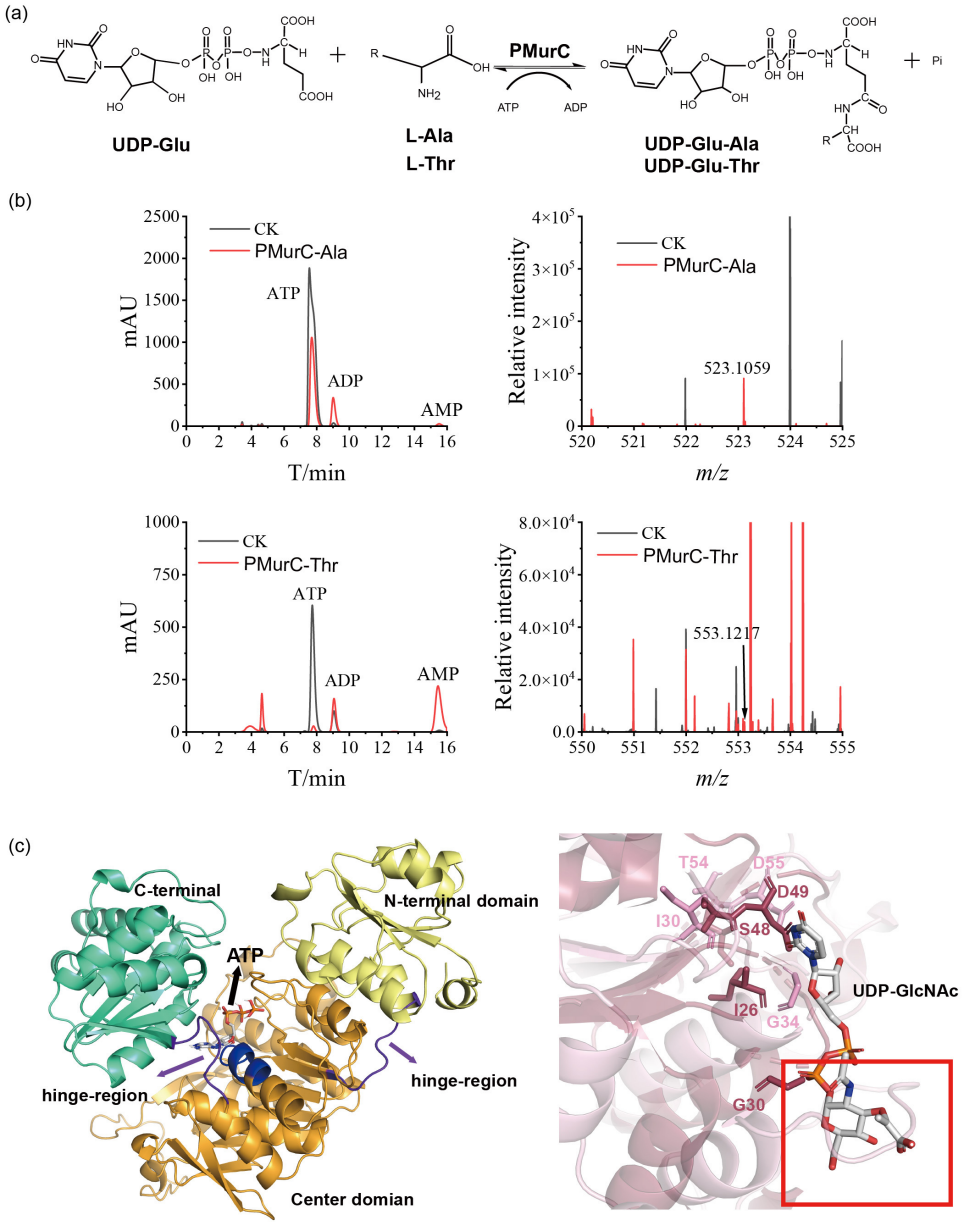
Activity and structural analysis of PMurC: **(a)** equation of reaction catalyzed by PMurC; **(b)** HPLC analysis of reaction buffer revealed reduction of ATP (left), and mass spectrometry analysis of reaction extracts revealed product, with peak at *m/z*=553.1217 (right); and **(c)** PMurC from *M. ruminantium* is in cartoon color with three functional domains, with conserved P-loop in blue color (left); conserved UDP-binding residues are shown as colored sticks in their corresponding proteins (PMurC, light pink; MurC (PDB: 1GQQ), raspberry), and obstacles are marked with red box, right. HPLC analyses were performed in triplicate for each sample. CK: control check group (enzymatic reaction system proceeds without enzyme addition).

Previous studies have indicated that the second amino acid in pseudomurein pentapeptides can be either threonine or alanine, depending on the methanogen species. For example, Leahy et al. (2010) reported that threonine is utilized in the synthesis of the pseudomurein peptide unit in *M. ruminantium*, whereas *M. fervidus* incorporates alanine (Renner-Schneck et al., 2015). Based on the study hypothesis, PMurC is proposed to catalyse the ligation of alanine or threonine to UDP-Glu in the presence of ATP, forming UDP-Glu-Ala or UDP-Glu-Thr (Figure 3a). To investigate substrate specificity, alanine and threonine were tested as substrates in enzymatic assays with purified PMurC from *M. ruminantium*. Consequently, ATP consumption and the production of UMP-Glu-Ala or UMP-Glu-Thr were detected. Product fragments corresponding to UMP-Glu-Ala (*m/z*: 524.1155 Da) and UMP-Glu-Thr (*m/z*: 554.1261 Da) were observed via LC-MS (Figure 3b). Additionally, tandem mass spectrometry confirmed the presence of these products (Supplementary Figure S4d). Notably, PMurC exhibited higher activity toward alanine than threonine, despite *M. ruminantium* utilizing threonine in its pentapeptide. We speculate that this may be because threonine has a side chain group, while alanine does not. During the catalytic reaction, the side chain group causes steric effects, resulting in lower catalytic activity of threonine compared to alanine.

The overall structure of PMurC from *M. ruminantium* is identical to that of PMurC from *M. fervidus* (PDB: 6VR7), which was resolved in a complex with UDP. Both structures are also similar to that of bacterial MurC (Supplementary Figure S8a). PMurC consists of three domains: an N-terminal domain for uridine binding, a central domain that provides an ATP-binding site featuring a conserved P-loop, and a C-terminal domain that accommodates amino acid substrates (Figure 3c, left and Supplementary Figure S6). Short loops connecting these domains likely function as hinge regions, similar to those in MurC, facilitating domain rearrangement from the apo to the closed conformation.

Molecular docking simulations revealed that the ATP-binding site is located at the interface between the central and N-terminal domains, specifically within the conserved P-loop (GTNGKTTT), wherein the histidine residue of the bacterial P-loop is replaced by Asn^116^ in all members of the putative PMurC family (Figure 3c and Supplementary Figure S6). The UDP-binding site is identical to that of MurC, involving conserved residues in the N-terminal domain. However, although the sugar moiety of UDP-MurNAc is deeply embedded within MurC, the corresponding region in PMurC is completely occluded by bulky residues (Figure 3c, right). This structural distinction enables PMurC to utilize uridine-linked peptides as substrates, in contrast to bacterial ligases that accommodate sugars. Additionally, PMurC contains a zinc-binding site composed of four conserved cysteines, which is present in all PMurC family proteins but absent in bacterial murein ligases; the function of this site remains unknown (Supplementary Figures S7, S8b). Although PMurC and MurC share high structural similarity in domain rearrangement, they do not perform the same biological function, as MurC cannot catalyse PMurC's reaction.

### 3.4 PMurE catalyses the ligation of lysine to UDP-Glu-Ala, producing UDP-Glu-Ala-Lys

**Figure 4:**
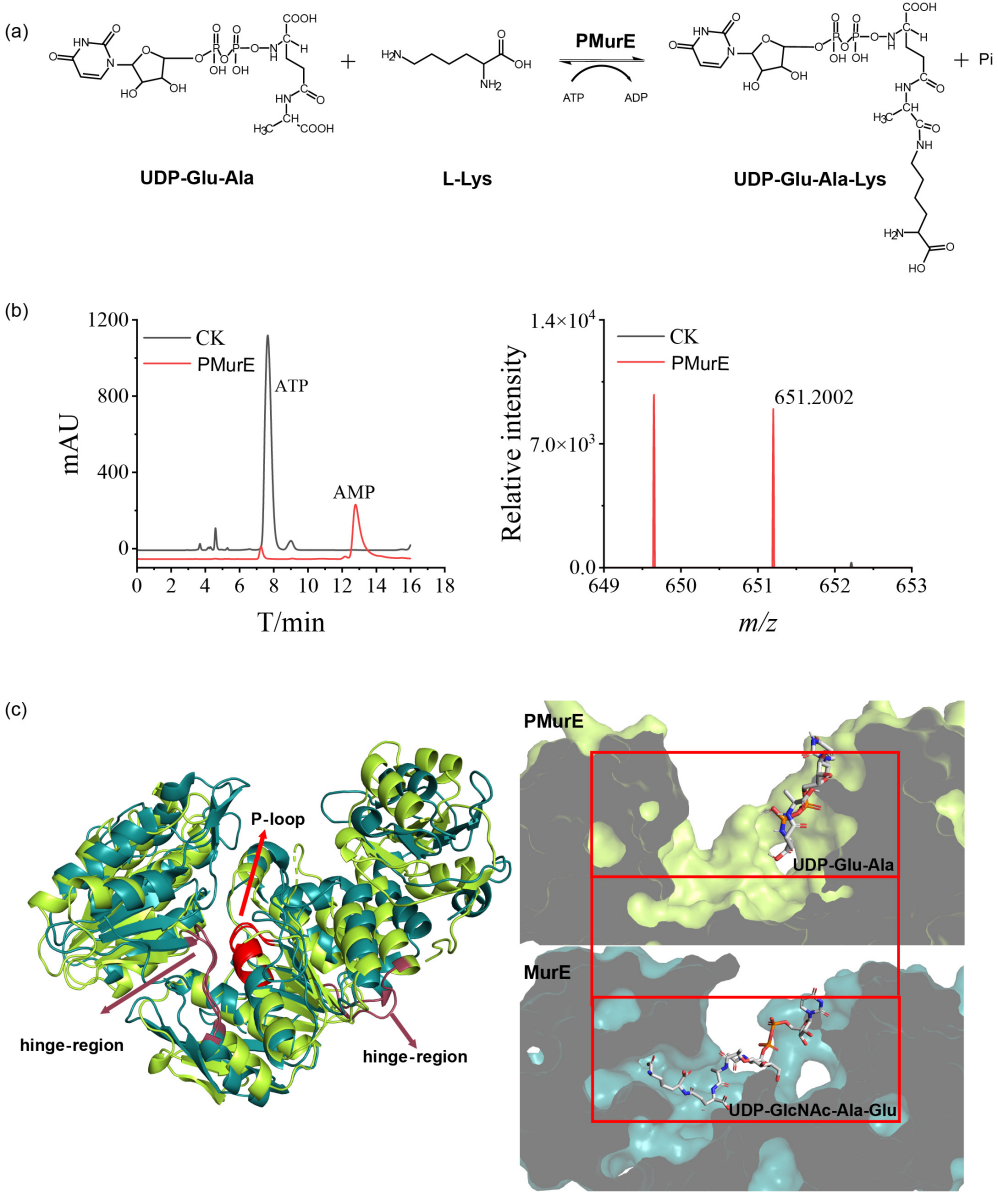
Activity and modeled structure of PMurE: **(a)** equation of reaction catalyzed by PMurE; **(b)** analysis of ATP hydrolysis capacity of PMurE for different amino acid substrates with HPLC (left), and high-resolution detection of substrate with different amino acid substrates (right); and **(c)** alignment between PMurE (PDB 6VR8, lemon color) and MurE [PDB 1E8C (Gordon et al., 2001), deep teal color], and hinge regions are raspberry-colored, and conserved P-loop is red-colored, left; zoomed-in view of binding pocket, right. HPLC analyses were performed in triplicate for each sample. CK: control check group (enzymatic reaction system proceeds without enzyme addition).

We hypothesized that PMurE is the key enzyme responsible for synthesizing UDP-Glu-Ala-Lys from UDP-Glu-Ala (Figure 4a). The structure of pMurE has been determined and is most similar to that of the bacterial peptide ligase MurE (Figure 4c, left) (Subedi et al., 2022). To confirm its biological function, we assayed the activity of purified PMurE from *M. fervidus* using chemically synthesized UMP-Glu-Ala as a substrate. Consequently, PMurE hydrolysed ATP to ADP, utilizing the released energy to catalyse the formation of UMP-Glu-Ala-Lys (*m/z*: 652.2105; Figure 4b). Tandem mass spectrometry further confirmed this product through fragments with *m/z* 303.1 and 325.9 (Supplementary Figure S4h). Similar to the Mur family, PMurE possesses conserved residues that form UTP- and ATP-binding sites at the interface between the central and N-terminal domains (Supplementary Figure S9). The substrate UDP-Glu-Ala occupies the UDP-binding site (PDB: 7UFP), which presents a continuous surface adjacent to the ATP-binding site, facilitating the formation of the uridine-acyl-phosphate intermediate (Supplementary Figure S10). Compared to PMurE, the MurE structure features a longer and narrower binding channel that accommodates the substrate, whereas PMurE has a shorter one (Figure 4c, right). These differences in binding patterns indicate that PMurE has a distinct catalytic function from MurE, as evidenced by MurE's inactivity with UDP-Glu-Ala as a substrate.

### 3.5 PMurC-D and PMurC-E are assumed to undergo similar conformational changes during the catalytic cycle

**Figure 5:**
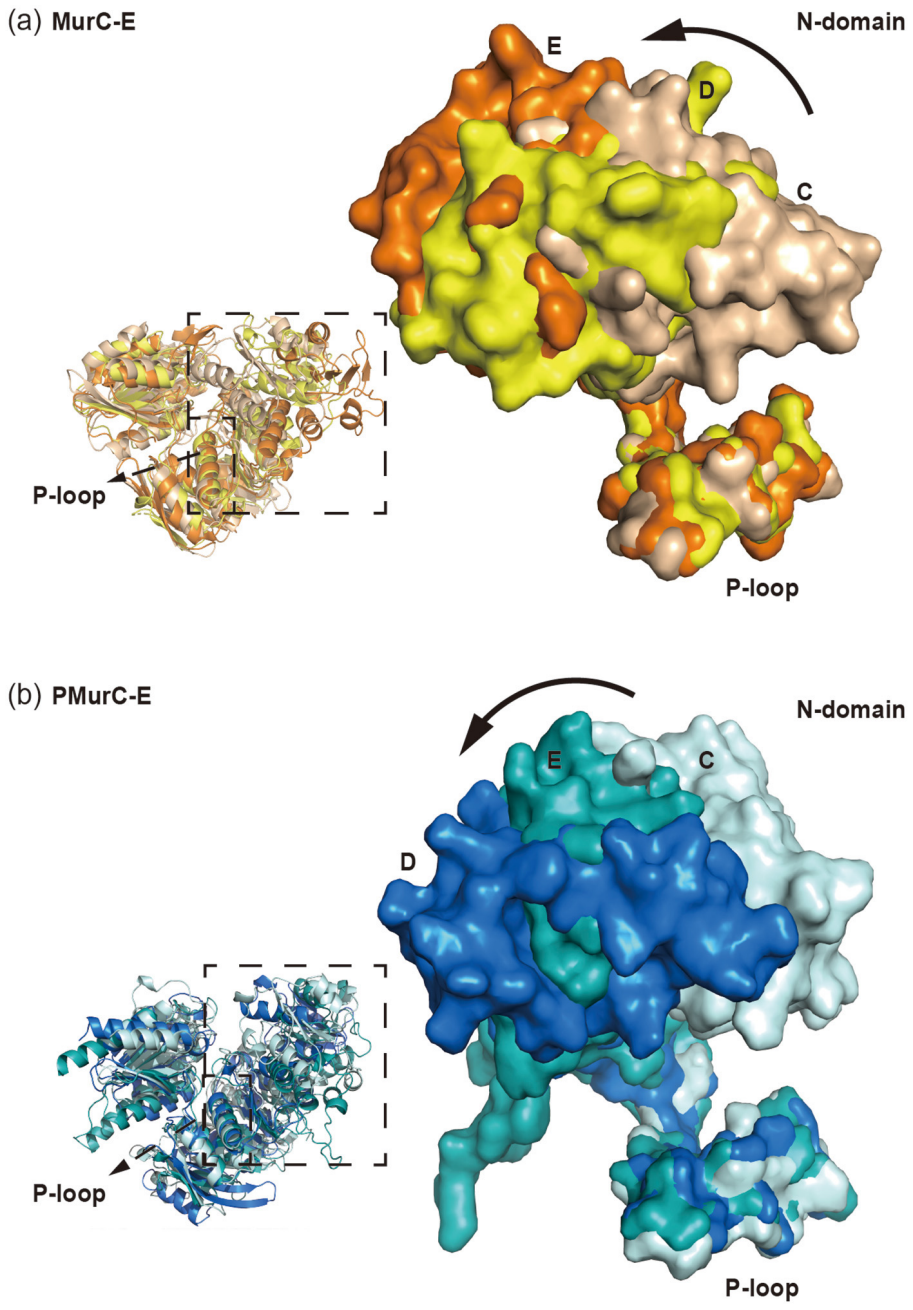
N-domain shift of MurC-E and PMurC-E: **(a)** superposition of MurC-E shows that N-domain is more open when substrates are longer (MurC, PDB: 1P31, pale yellow; MurE, PDB: 1E8C, light brown; MurD, PDB: 2UAG, orange); zoomed-in view of N-domain is shown as surface; **(b)** superposition of PMurC-E shows that N-domain is more open when substrates are longer (PMurC, PDB: 6VR7, pale cyan; PMurE, PDB: 6VR8, teal, PMurD, AlphaFold2-predicted model blue); zoomed-in view of N-domain is shown as surface.

In the catalytic cycle of Mur ligases, substrate binding induces a conformational change from an open to a closed state, wherein the amino acid and UDP-linked sugar are positioned adjacent to ATP to facilitate the reaction (Smith, 2006; Mol et al., 2003). This substrate-induced domain rearrangement is also conserved in PMur ligases. Furthermore, relative to the structures of PMurC, PMurE, and PMurD, the N-terminal domain of PMurC-E shifts outward, progressively enlarging the substrate-binding pocket as UDP-linked peptides elongate during biosynthesis (Figure 5).

### 3.6 Unknown proteins of the archaeal pseudomurein biosynthesis pathway

Based on the primary structural analysis, PMurA is predicted to catalyse the phosphorylation of glutamate in the presence of ATP (Supplementary Table S1). PMurA from *M. ruminantium* was heterologously expressed in *E. coli* and subjected to enzymatic assays using glutamate as the substrate. The results indicated that PMurA degrades ATP to ADP and AMP, akin to an ATPase (Supplementary Figure S11a). However, the predicted product, Glu-Pi (Glu-Pi; *m/z* 227.0194) was not detected using LC-MS, possibly due to limitations in the detection method or because PMurA does not mediate glutamate phosphorylation. Further refinement of target gene identification is required to elucidate the initial step of pentapeptide biosynthesis.

As previously noted, two MurD homologs (PMurD1 and PMurD2) are present in pseudomurein-producing methanogens (Figure 1b). Sequence analysis indicates that they share 20%−30% identity with MurD and possess a conserved ATP-binding domain and a highly conserved P-loop (Supplementary Figure S12). PMurD demonstrated significant ATP hydrolysis activity (Supplementary Figures S11b, c); however, the expected product UMP-Glu-Ala-Lys-Ala was not detected (Mw: 764.25 Da). The function of PMurD remains undetermined, potentially due to an additional acyl group on the lysine residue of the chemically synthesized precursor UMP-Glu-Ala-Lys-Ala.

## 4 Conclusions

Pseudomurein is a key component of methanogen cell walls. It is well-established that pseudomurein biosynthesis follows a process similar to that of bacterial murein (peptidoglycan). Most homologous genes in the murein biosynthesis pathway have been identified in methanogens. However, limited information is available on murein biosynthesis owing to the stringent growth requirements of archaea and the unavailability of precursors. In this study, we chemically synthesized UMP-linked peptide substrates and identified three key enzymes of pseudomurein biosynthesis: PMurB, which is exclusive to archaea and catalyses the conjugation of UDP-Glu and Pi, forming UDP; PMurC, which adds alanine or threonine to UDP-Glu, yielding UDP-Glu-Ala (or UDP-Glu-Thr); and PMurE, which ligates lysine to the PMurC product. To our knowledge, this study is the first report on the identification of the biological functions of PMurB, PMurC, and PMurE that describes the principal steps of the peptide chain biosynthesis pathway. Although the complete pathway remains to be enzymatically characterized, the study findings help in outlining future studies for clarifying the remaining steps of the pathway.

The structures of PMur and bacterial Mur ligases are nearly identical, underscoring their close evolutionary relationship. However, they catalyse entirely different reactions. Molecular docking simulations indicate that the larger sugar moiety of the murein intermediate cannot be accommodated by PMur, potentially explaining the substrate specificity divergence between archaeal and bacterial ligases.

Cell walls are key targets of bacterial growth inhibitors and biotechnology applications. Methanogens cause losses in livestock yield by converting animal feed to methane instead of milk or meat. Our findings improve the understanding of pseudomurein biosynthesis, with implications for methanogen biotechnology, and a better understanding of Pmur enzyme mechanisms may aid the development of inhibitors of methanogen growth in animal digestive systems. Further elucidation of the pseudomurein biosynthesis pathway should facilitate the identification of cell wall synthesis inhibitors, targeting methane emission and bioenergy.

## Data Availability

The original contributions presented in the study are included in the article/Supplementary material, further inquiries can be directed to the corresponding authors.
