# Supplementary material for: Enzymatic activities of proteins encoded by PmurB, PmurC, and PmurE involved in methanogen pseudomurein biosynthesis
Source: Front Microbiol. 2026 Mar 4;17:1766147. doi: 10.3389/fmicb.2026.1766147 (PMC12996149; doi:10.3389/fmicb.2026.1766147)
Supplement: Supplementary file 1 [file Data_Sheet_1.docx]

**Definitive list of abbreviations**

**Abbreviations Full Form Description**

Mur Murein ligase Bacterial murein ligase

PMur Pseudomurein murein ligase Archaea pseudomurein murein ligase

CK Control check Control check group

DCC Dicyclohexylcarbodiimide Chemical reagent

HOBt Hydroxybenzotriazole Chemical reagent

Ala Alanine Amino acid

AMP Adenosine monophosphate Nucleotide

ATP Adenosine triphosphate Nucleotide energy carrier

ADP Adenosine diphosphate Nucleotide

Da Dalton Unit of molecular mass

Glu Glutamate Amino acid

GlcNAc N-acetylglucosamine Sugar component in peptidoglycan

HPLC: High-performance liquid chromatography Analytical technique

i-PrOH Isopropanol Solvent

LC-MS Liquid chromatography–mass spectrometry Analytical technique

Lys Lysine Amino acid

MurNAc N-acetylmuramic acid Sugar component in peptidoglycan

NAcTalNA N-acetyltalosaminuronic acid Sugar component in pseudomurein

Pi-Glu Phosphoglutamate Phosphorylated glutamate

PMurA–E Pseudomurein Mur ligases A–E Pseudomurein synthesis enzymes

Thr Threonine Amino acid

t-BuOH tert-Butanol Solvent

LCT Thin-layer chromatography Analytical technique

UDP Uridine diphosphate Nucleotide

UDP-Glu UDP-glutamate Activated glutamate intermediate

UDP-Glu-Ala UDP-glutamyl-alanine Peptide intermediate

UDP-Glu-Ala-Lys UDP-glutamyl-alanyl-lysine Peptide intermediate

UDP-Glu-Thr UDP-glutamyl-threonine Peptide intermediate

UMP-Glu UMP-glutamate Analogue intermediate

UMP-Glu-Ala UMP-glutamyl-alanine Analogue intermediate

UMP-Glu-Ala-Lys-Ala UMP-glutamyl-alanyl-lysyl-alanine Analogue intermediate

UMP-Glu-Thr UMP-glutamyl-threonine Analogue intermediate

**Supplementary Information**

**Table S1.** *M. ruminantium* M1 pseudomurein peptide unit precursor synthesis.

| **Gene** | **Enzyme** | **Strain** | **Accession Number** | **Pfam** |
| --- | --- | --- | --- | --- |
| MRU-RS05605 | PMurA | *M. ruminantium* M1 | WP_143714313.1 |  |
| MRU-RS05600 | PMurB | *M. ruminantium* M1 | WP_012955916.1 | 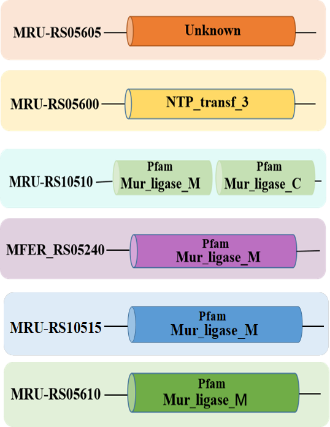 |
| MRU-RS10510 | PMurC | *M. ruminantium* M1 | WP_048812528.1 |  |
| MRU-RS05240 | PMurE | *M. ruminantium* M1 | WP_012955843.1 |  |
| MRU-RS10515 | PMurD1 | *M.* *ruminantium* M1 | WP_012956890.1 |  |
| MRU-RS05610 | PMurD2 | *M.* *ruminantium* M1 | WP_012955918.1 |  |

**Table S2.** Information related to the construction of vectors for key enzymes of the pseudomurein peptide unit precursor synthesis pathway.

| **Source strain** | **Expression strain** | **Plasmid** | **Antibiotic** | **Protein tag** |
| --- | --- | --- | --- | --- |
| *Methanobrevibacter ruminantium* | PMurA-BL21(DE3) | pET-28a | Kan | His-tag |
| *Methanobrevibacter ruminantium* | PMurB-BL21(DE3) | pET-28a | Kan | His-tag |
| *Methanobrevibacter ruminantium* | PMurC-BL21(DE3) | pET-28a | Kan | His-tag |
| *Methanobrevibacter ruminantium* | PMurE1-BL21(DE3) | pET-28a | Kan | His-tag |
| *Methanobrevibacter ruminantium* | PMurD1-BL21(DE3) | pET-28a | Kan | His-tag |
| *Methanobrevibacter ruminantium* | PMurD2-BL21(DE3) | pGEX-6p | Amp | His-tag |
| *Methanothermus fervidus* DSM 2088 | PMurE2-BL21 Star (DE3) | pET-28a | Kan | His-tag |

**Table S3.** Information about gene clusters that synthesise pseudomurein peptide units.

| **Source strain** | **Name of protein** | **Gene symbol of key genes of the pseudourethane peptide unit synthesis pathway** |
| --- | --- | --- |
| *Methanobrevibacter ruminantium* M1 | PMurA | MRU_RS05605 |
|  | PMurB | MRU_RS05600 |
|  | PMurC | MRU_RS10510 |
|  | PMurD1 | MRU_RS05240 |
|  | PMurD2 | MRU_RS10515 |
|  | PMurE | MRU_RS05610 |
| *Methanothermus fervidus* DSM 2088 | PMurA | MFER_RS01690 |
|  | PMurB | MFER_RS01685 |
|  | PMurC | MFER_RS01695 |
|  | PMurD1 | MFER_RS03870 |
|  | PMurD2 | MFER_RS01700 |
|  | PMurE | MFER_RS01715 |
| *Methanopyrus kandleri* AV19 | PMurA | MK_RS09180 |
|  | PMurB | MK_RS06405 |
|  | PMurC | MK_RS04015 |
|  | PMurD1 | MK_RS04000 |
|  | PMurD2 | MK_RS05265 |
|  | PMurE | MK_RS04900 |
| *Methanobrevibacter smithii* ATCC 35061 | PMurA | MSM_RS00570 |
|  | PMurB | MSM_RS00565 |
|  | PMurC | MSM_RS05760 |
|  | PMurD1 | MSM_RS04400 |
|  | PMurD2 | MSM_RS00575 |
|  | PMurE | MSM_RS07875 |
| *Methanothermobacter marburgensis* str. Marburg | PMurA | MTBMA_RS04490 |
|  | PMurB | MTBMA_RS04485 |
|  | PMurC | MTBMA_RS05505 |
|  | PMurD1 | MTBMA_RS04495 |
|  | PMurD2 | MTBMA_RS04450 |
|  | PMurE | MTBMA_RS05505 |
| *M. thermautotrophicus* str*.* Delta H | PMurA | MTH_RS02455 |
|  | PMurB | MTH_RS02450 |
|  | PMurC | MTH_RS02460 |
|  | PMurD1 | MTH_RS09990 |
|  | PMurD2 | MTH_RS02470 |
|  | PMurE | MTH_RS03440 |
| *Methanobacterium paludis* | PMurA | MSWAN_RS01900 |
|  | PMurB | MSWAN_RS01895 |
|  | PMurC | MSWAN_RS02135 |
|  | PMurD1 | MSWAN_RS02140 |
|  | PMurD2 | MSWAN_RS01905 |
|  | PMurE | MSWAN_RS02145 |
| *Methanobrevibacter milerae* | PMurA | SM9_RS04645 |
|  | PMurB | SM9_RS04035 |
|  | PMurC | SM9_RS00535 |
|  | PMurD1 | SM9_RS00530 |
|  | PMurD2 | SM9_RS04640 |
|  | PMurE | SM9_RS03310 |
| *Methanosphaera stadtmanae* DSM 3091 | PMurA | MSP_RS06395 |
|  | PMurB | MSP_RS06400 |
|  | PMurC | MSP_RS06005 |
|  | PMurD1 | MSP_RS06010 |
|  | PMurD2 | MSP_RS06385 |
|  | PMurE | MSP_RS06015 |

**Table S4.** The key enzyme substrate and product analogue molecules of the pseudopeptidoglycan peptide unit synthesis pathway, with information on their fragment ions produced in the MS2 assay.

| **Compound** | | **Mw** | **Chemical formula** | | **Structural formula** | **Fragment of MS2 (*m/z*)** |
| --- | --- | --- | --- | --- | --- | --- |
| Pi-Glu | 227.01949 | | | C_5_H_10_NO_7_P |  | 132.04226  96.99288 |
| UMP-Glu-Ala | 524.11557 | | | C_17_H_25_N_4_O_13_P |  | 294.06169  186.07718 |
| UMP-Glu-Thr | 554.12614 | | | C_18_H_27_N_4_O_14_P |  | 421.08863  404.08644 |
| UMP-Glu-Ala-Lys | 652.20121 | | | C_24_H_39_N_6_O_15_ |  | 327.08315  304.163.44 |

**Table S5.** Information on PMur and Mur for gene blast.

| **Enzyme** | **Organism** | **Accession Number** |
| --- | --- | --- |
| PMurB | *M. ruminantium* M1 | WP_012955916.1 |
|  | *M. fervidus* | WP_013413415.1 |
| MurU | *Pseudomonas putida* BIRD-1 | WP_012955843.1 |
|  | *Caulobacter sp.* | WP_291876948.1 |
|  | *Brevundimonas kwangchunensis* | WP_343790889.1 |
| Transferases | *Salmonella enterica* | 1IIM_A |
|  | *Pseudomonas aeruginosa* | WP_003105518.1 |
|  | *Escherichia coli* | WP_000676056.1 |
|  | *Bacillus anthracis* | WP_000676166.1 |
| PMurC | *M. ruminantium* M1 | WP_048812528.1 |
|  | *M. fervidus* | WP_013413417.1 |
| MurC | *Haemophilus influenzae* | WP_005693453.1 |
|  | *Escherichia coli* | WP_001096049.1 |
|  | *Staphylococcus aureus* | WP_000150169.1 |
|  | *Neisseria meningitidis serogroup* C | WP_002222038.1 |
|  | *Stenotrophomonas maltophilia* | WP_005408080.1 |
| PMurE | *M. fervidus* | WP_013413839.1 |
| MurE | *Bacillus cereus* | WP_000766313.1 |
|  | *Escherichia coli* | GCA_900448195.1 |
|  | *Thermotoga maritima* | WP_004082936.1 |
|  | *Staphylococcus aureus* | WP_000340126.1 |
| PMurD1 | *M. ruminantium* M1 | WP_012956890.1 |
| PMurD2 | *M. ruminantium* M1 | WP_012955918.1 |
| MurD | *Escherichia coli* | HCP3975063.1 |
|  | *Pseudomonas aeruginosa* PAO1 | WP_003103104.1 |
|  | *Haemophilus influenzae* | WP_005693453.1 |
|  | *Streptococcus* | WP_000849681.1 |

**Figure S1.** High-resolution primary mass spectrometry detection of UMP-amino acid derivatives: (a) high-resolution mass spectrometry of UMP-Glu; (b) high-resolution mass spectrometry of UMP-Glu-Ala; and (c) high-resolution mass spectrometry of UMP-Glu-Ala-Lys(like).


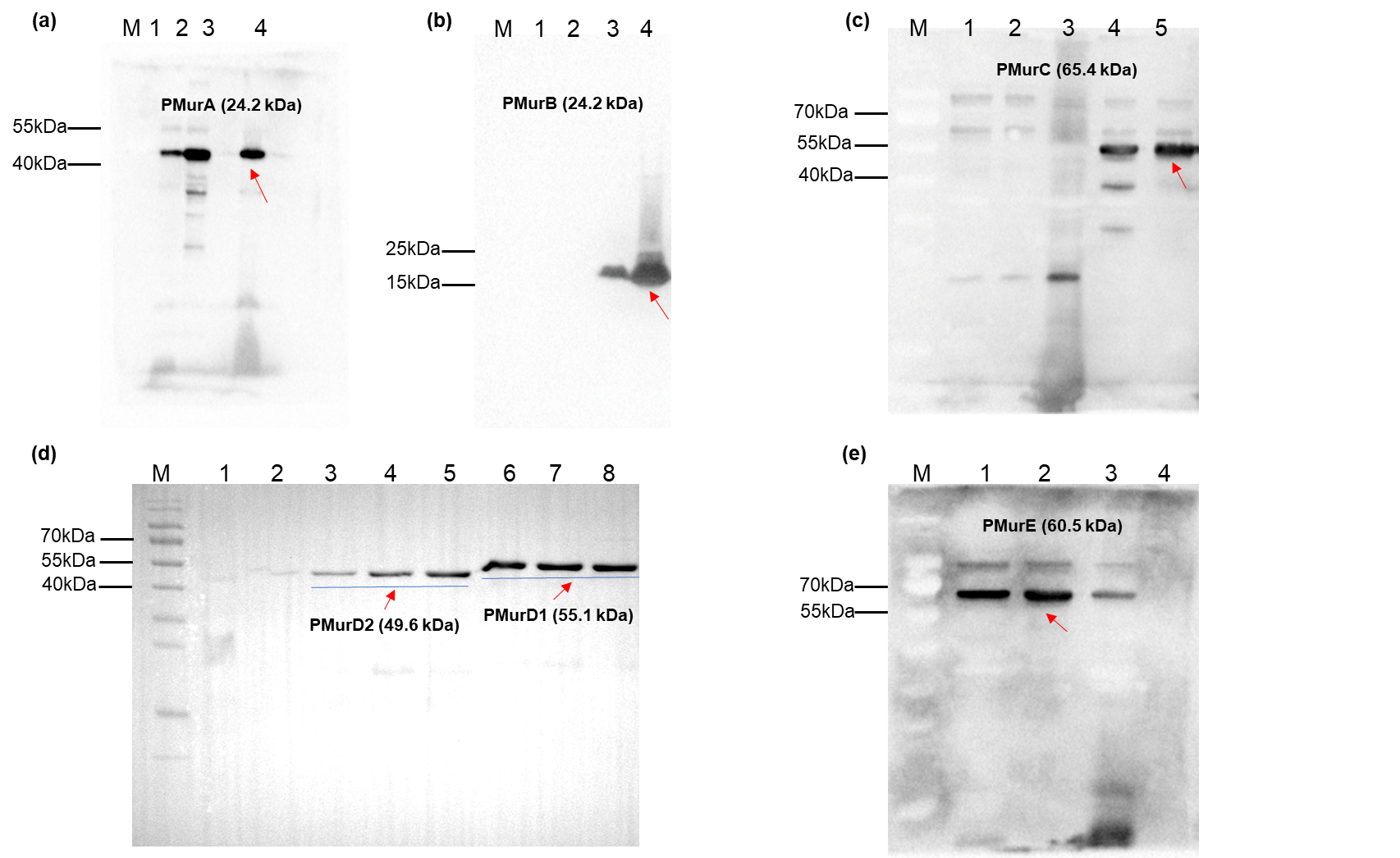


**Figure S2.** Western blot of PMurA-E from *M. ruminantium* M1 and *M. fervidus*. (**a**) Western blot of the PMurA protein. Line 1, vector control from *E. coli* BL21 (DE3). Lines 2-3, crude extract from *E. coli* BL21 (DE3) induced with 1 mM IPTG and grown at 37℃ for 3 h. Line 4, purification of the recombinant PMurA protein. (**b**) Western blot of the PMurB protein. Lines 1-2, vector control from *E. coli* BL21 (DE3). Line 3, crude extract from *E. coli* BL21 (DE3) induced with 1 mM IPTG and grown at 25℃ for 1 h. Line 4, purification of the recombinant PMurB protein. (**c**) Western blot of the PMurC protein. Lines 1-3, vector control from *E. coli* BL21 (DE3). Line 4, crude extract from *E. coli* BL21 (DE3) induced with 1 mM IPTG and grown at 30℃ for 3 h. Line 5, purification of the recombinant PMurC protein. (**d**) Western blot of the PMurD1 and PMurD2 proteins. Line 1-2, vector control from *E. coli* BL21 (DE3). Lines 3-5, purification of the recombinant PMurD2 protein. Lines 6-8, purification of the recombinant PMurD1 protein. (**e**) Line 4, vector control from *E. coli* BL21 (DE3). Lines 1-2, crude extract from *E. coli* BL21 (DE3) induced with 1 mM IPTG and grown at 37℃ for 1 h. Line 3, purification of the recombinant PMurE protein. M: prestained protein size markers. Target protein is shown in red arrow.


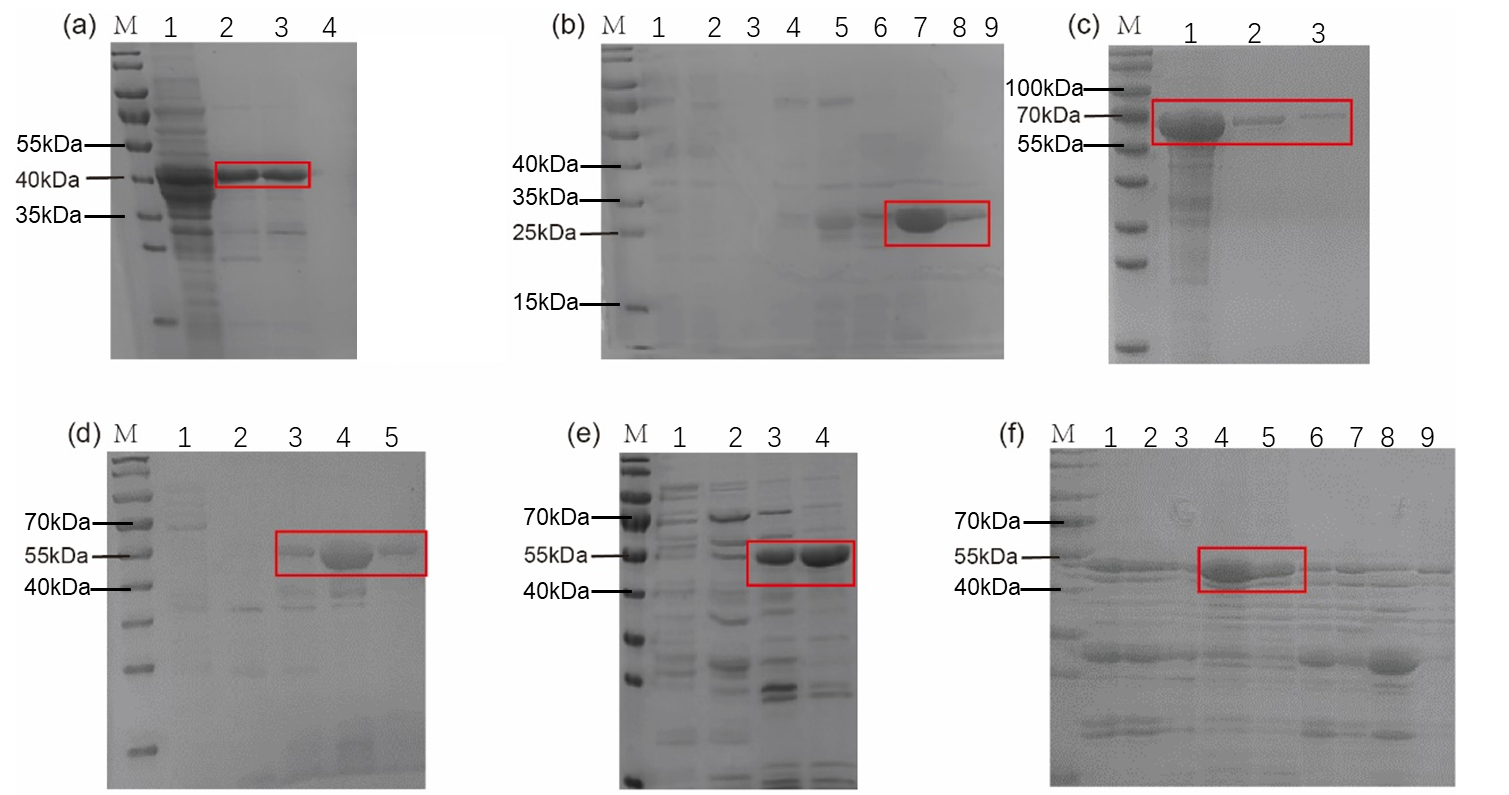


**Figure S3.** SDS–PAGE electrophoresis used for purification of PMurA-E: (**a**) Purification of the recombinant PMurB protein; Line 1, Supernatant. Line 2, protein solubilized in His-bind washing buffer. Line 3, protein solubilized in His-bind elution buffer. (**b**) Purification of the recombinant PMurA protein; Line 1, Supernatant. Lines 2-4, protein solubilized in His-bind binding buffer. Lines 5-6, protein solubilized in His-bind washing buffer. Lines 7-9, protein solubilized in His-bind elution buffer. (**c**) Purification of the recombinant PMurC protein; Line 1, protein solubilized in His-bind washing buffer. Lines 2-3, protein solubilized in His-bind elution buffer. (**d**) Purification of the recombinant PMurE protein; Line 1, Supernatant. Line 2, protein solubilized in His-bind binding buffer. Line 3, protein solubilized in His-bind washing buffer. Lines 4-5, protein solubilized in His-bind elution buffer. (**e**) Purification of the recombinant PMurD1 protein; Line 1, Supernatant. Line 2, protein solubilized in His-bind binding buffer. Line 3, protein solubilized in His-bind washing buffer. Line 4, protein solubilized in His-bind elution buffer. (**f**) Purification of the recombinant PMurD2 protein. Line 1, Supernatant. Line 2-3, protein solubilized in His-bind binding buffer, Line 4, protein solubilized in His-bind washing buffer. Lines 5-9, protein solubilized in His-bind elution buffer. M: Prestained protein size markers. Target protein is shown in red box.


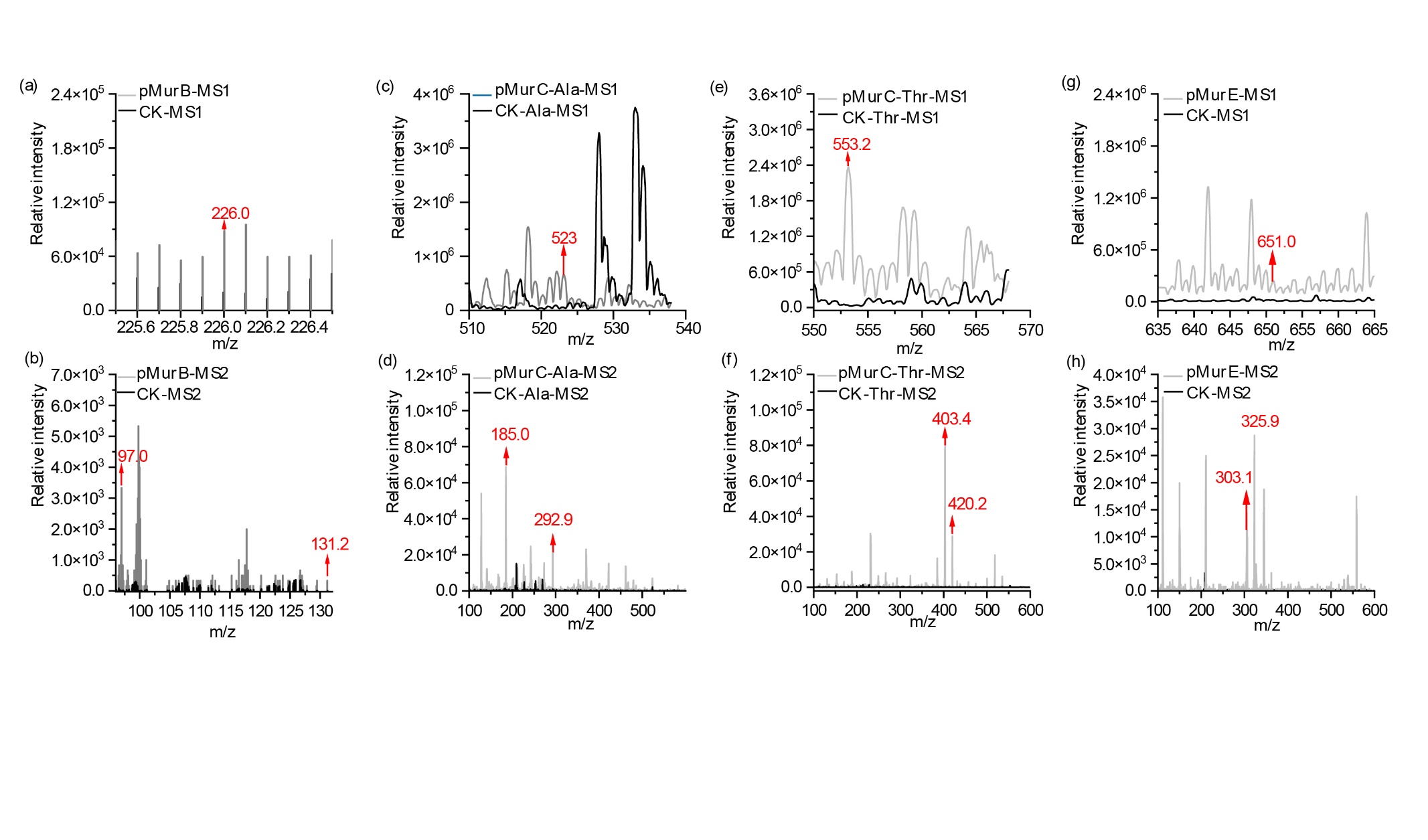


**Figure S4.** Pseudomurein peptide ligase product detection: (a-b) MS1 and MS2 assays of PMurB enzyme-catalysed products; (c-d) MS1 and MS2 assays of PMurC enzyme-catalysed products that used Ala as substrate; (e-f) MS1 and MS2 assays of PMurC enzyme-catalysed products that used Thr as substrate; and (g-h) MS1 and MS2 assays of PMurE enzyme-catalysed products.


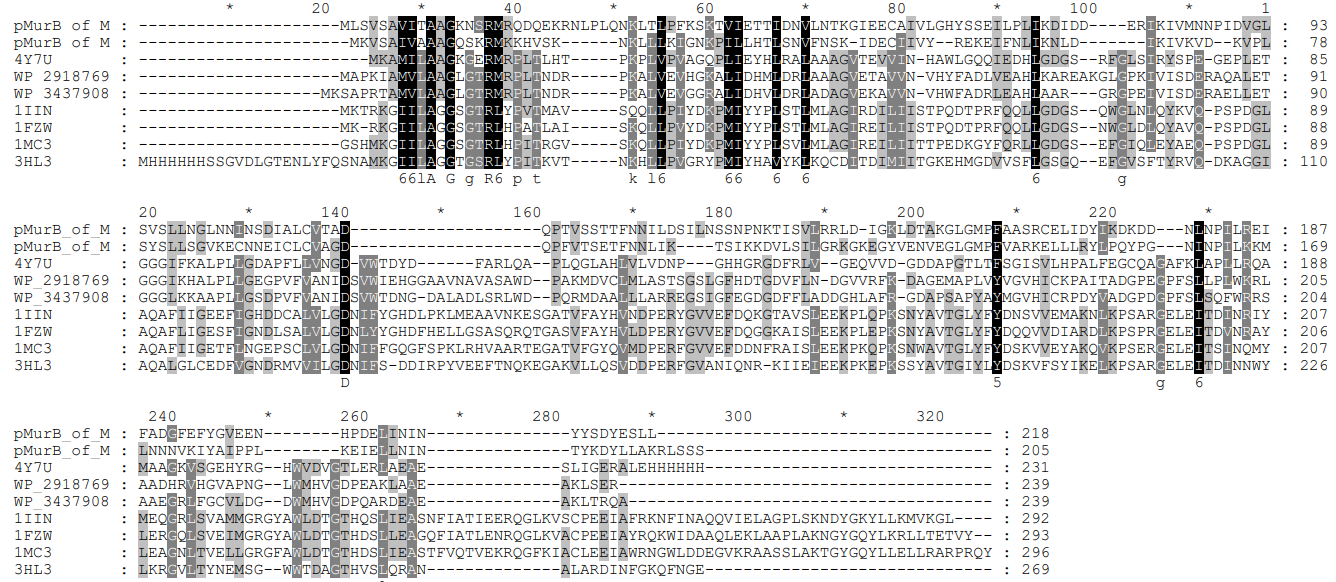


**Figure S5.** Sequence alignment of PMurB, MurU, and different transferases from bacterial species. First two sequences are of pMurB from *M. ruminantium* M1 and *M. fervidus* DSM 2088, respectively, next three sequences are of MurU downloaded from NCBI, and final four sequences are of known NTP transferase. Highly conserved residues are marked in black. Moreover, results show that they all have conserved UTP-binding domain. More detailed information on sequences is provided in Table S5.


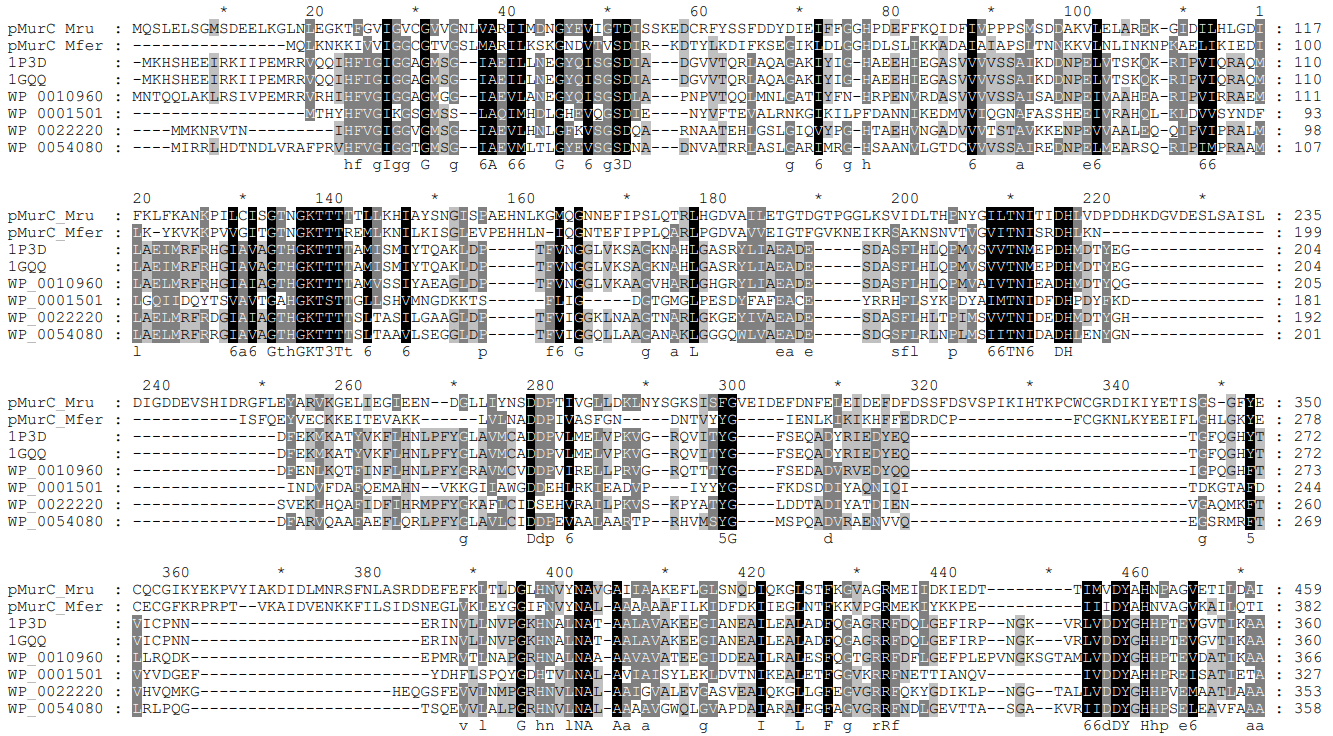


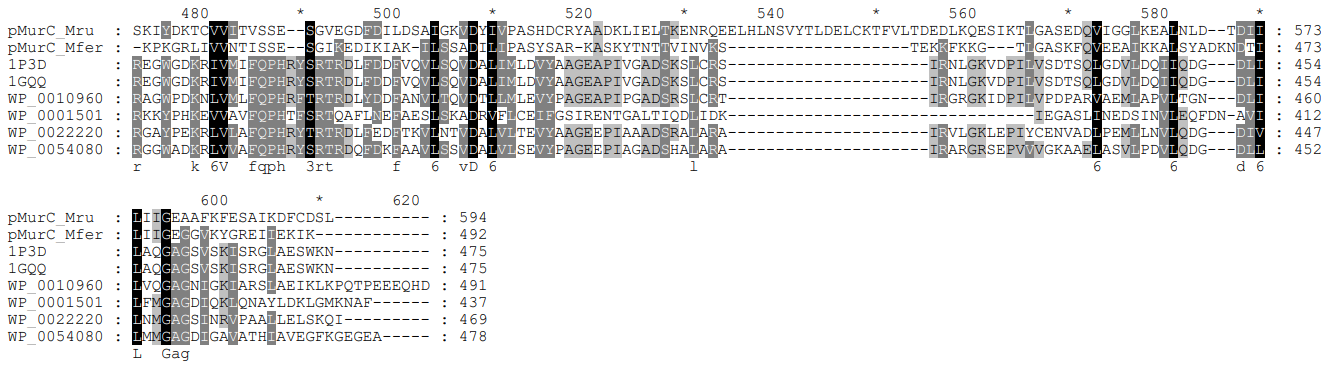


**Figure S6.** Sequence alignment of PMurC and different bacterial MurC. First two sequences are PMurC from *M. ruminantium* M1 and *M. fervidus* DSM 2088, respectively; next five sequences of bacterial MurC were downloaded from NCBI. Highly conserved residues are marked in black. More detailed information on sequences is provided in Table S5.


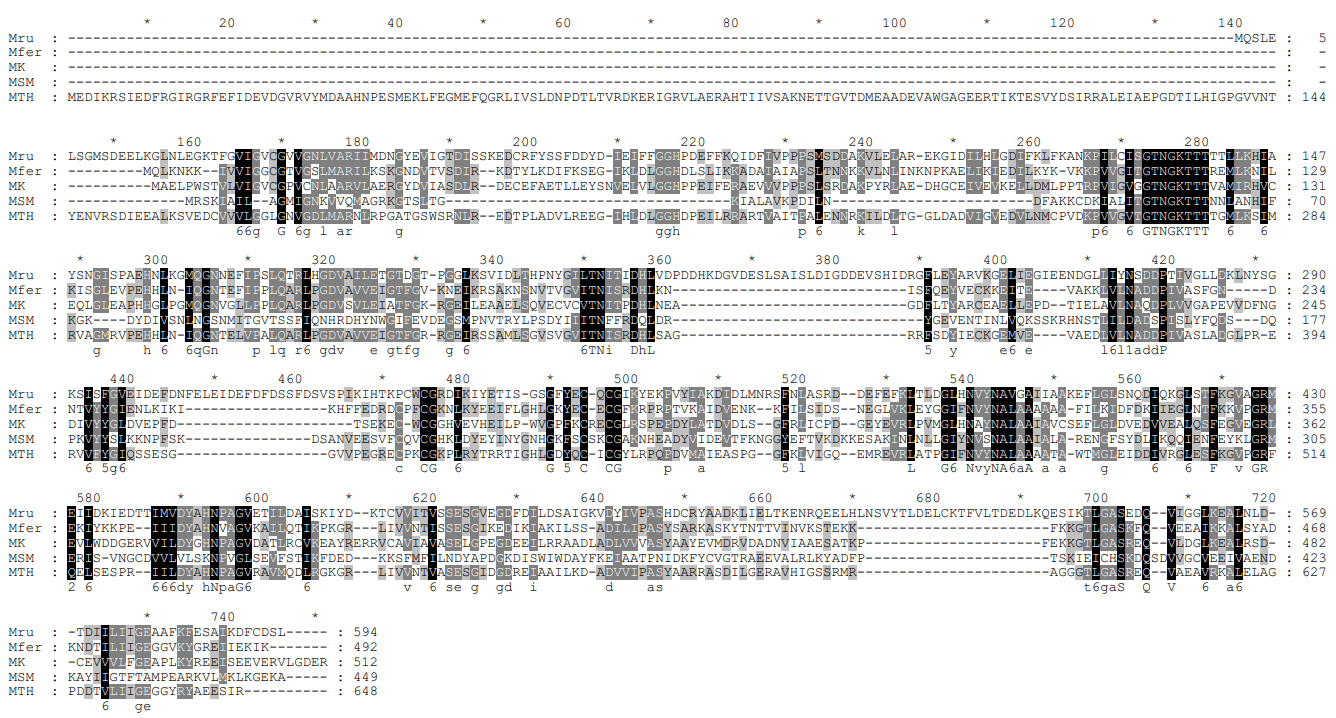


**Figure S7.** Sequence alignment of PMurC in different methanogens. Mru: *Methanobrevibacter ruminantium* M1; Mfer: *Methanothermus fervidus* DSM 2088; MK: *Methanopyrus kandleri* AV19; MSM: *Methanobrevibacter smithii* ATCC 35061; MTH: *M. thermautotrophicus* str. Delta H. More detailed information on sequences is provided in Table S5.


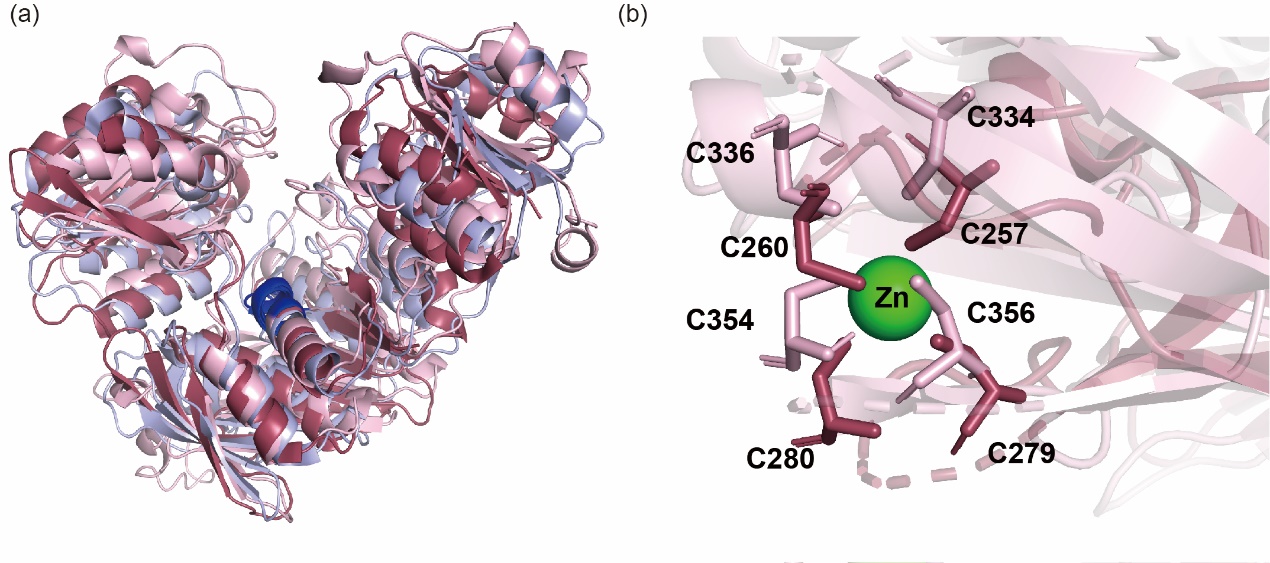


**Figure S8.** Structure alignment of PMurC and bacterial MurC: (a) all structures are shown as cartoons; PMurC from *M. ruminantium* M1, PMurC from *M. fervidus* DSM 2088 (PDB: 6VR8), and MurC from *Haemophilus influenzae* (PDB: 1GQQ) are light pink-, light blue-, and raspberry-coloured, respectively; conserved ATP-binding domains are coloured in blue; (b) Zn^+^ binding site is shown as sticks and labelled.


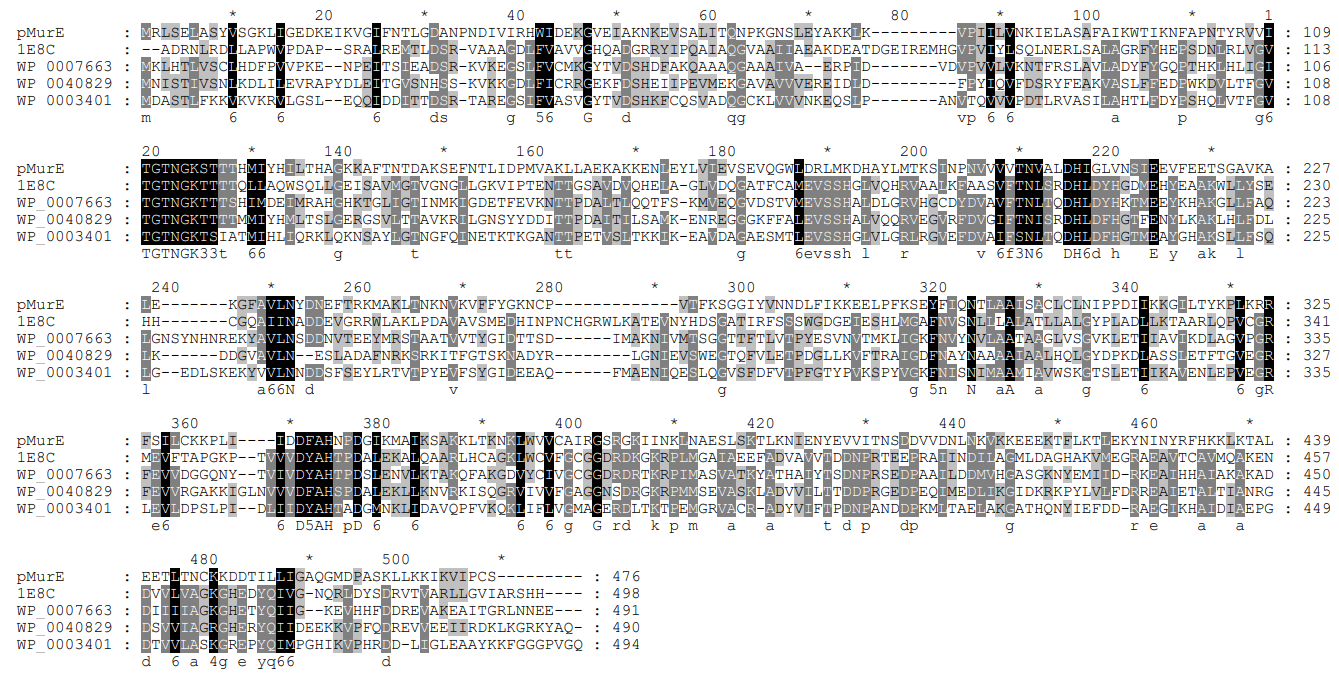


**Figure S9.** Sequence alignment of PMurE and different bacterial MurE. First two sequences are PMurE from *M. ruminantium* M1 and *M. fervidus* DSM 2088, respectively; next four sequences of bacterial MurE were downloaded from NCBI. Highly conserved residues are marked in black. More detailed information on sequences is provided in Table S5.


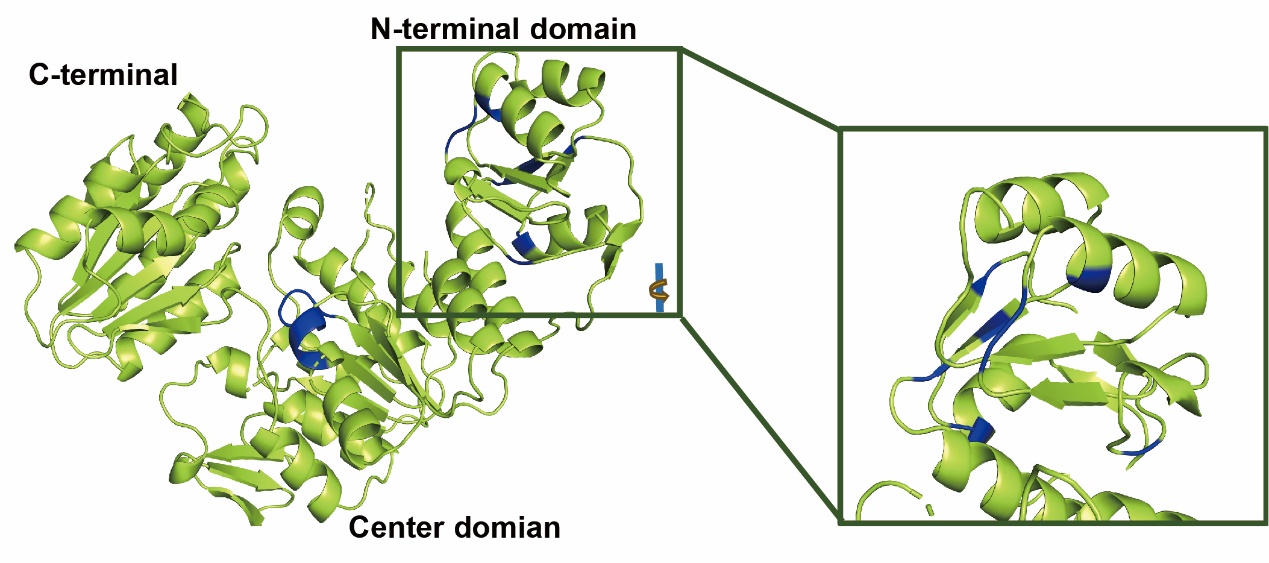


**Figure S10.** Structure of PMurE. Conserved UTP- and ATP-binding residues are coloured in blue.


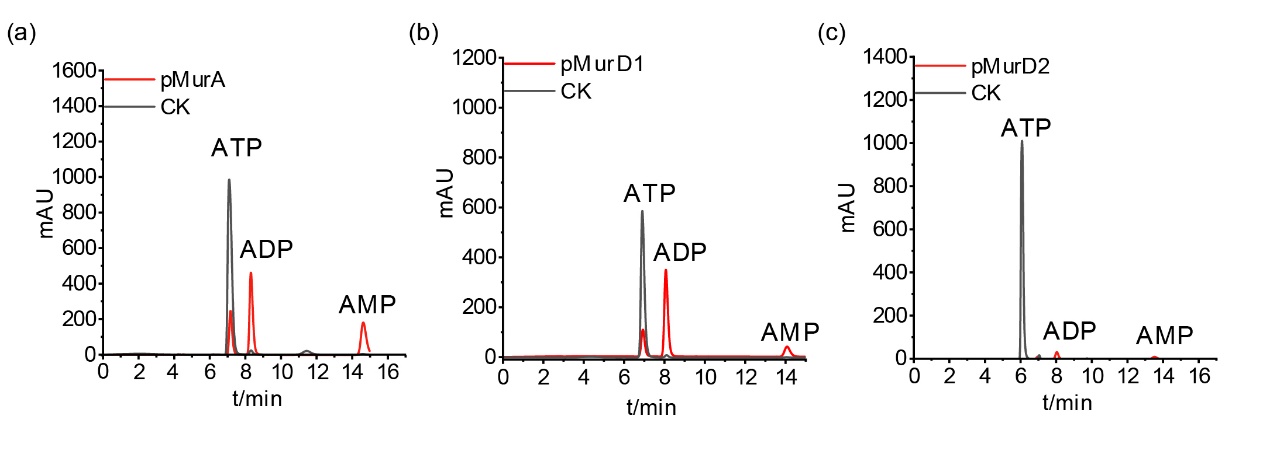


Figure S11. ATP activity assay of PMurA and PMurD1.


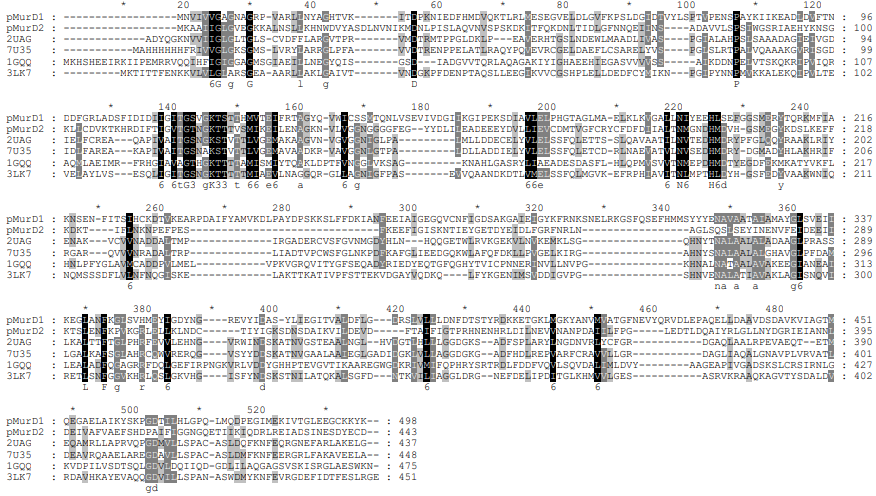


**Figure S12.** Sequence alignment of PMurD1 and D2 and different bacterial MurD. First two sequences are PMurD1 and D2 from *M. ruminantium* M1; next four sequences of bacterial MurD were downloaded from NCBI. Highly conserved residues are marked in black. More detailed information on sequences is provided in Table S5.
